# Editorial: Pediatric gastroenterology and visualizing the digestive tract

**DOI:** 10.3389/fped.2026.1877072

**Published:** 2026-06-04

**Authors:** Samuel Bitton, Kenneth Ng, Muhammad A. Khan

**Affiliations:** 1 Division of Pediatric Gastroenterology, Hepatology and Nutrition, Northwell Health, Cohen Children’s Medical Center, New Hyde Park, NY, United States; 2 Division of Pediatric Gastroenterology, Hepatology, and Nutrition, Department of Pediatrics, Johns Hopkins University School of Medicine, Baltimore, MD, United States; 3 Division of Gastroenterology, Hepatology and Nutrition, Nationwide Children’s Hospital, Columbus, OH, United States

**Keywords:** artificial intelligence - AI, confocal endomicroscopy, EndoFLIP (endoscopic functional lumen imaging probe), enteroscopy, ERCP, EUS (endoscopic ultrasound), transnasal endoscopy, video capsule endoscopy (VCE)

The landscape of pediatric gastroenterology stands at a technological crossroads, where innovation reshapes how we diagnose and treat digestive, liver, and nutritional disorders in children. Historically we have been constrained by the invasiveness of procedures, limits in device sizes, and a relative paucity of pediatric-specific data, but now the field is evolving toward approaches which are less invasive, more precise, and increasingly data-driven. These advances are redefining how clinicians visualize, interpret, and treat children with gastrointestinal diseases.

The contributions gathered in this Research Topic reflect four defining trends reshaping the field: minimally invasive endoscopy, diagnostic precision, advances in interventional endoscopy, and artificial intelligence integration. Together, these themes capture both the current state of pediatric endoscopy and its future trajectory.

## Minimally invasive endoscopy

Minimally invasive approaches are expanding the clinician's window into the gastrointestinal tract while reducing the physical and emotional burden on pediatric patients. Technologies such as video capsule endoscopy (VCE) and transnasal endoscopy (TNE) illustrate a broader shift toward improving tolerability without compromising diagnostic yield. Rojas et al. provided an in-depth review of video capsule endoscopy, while Granot et al. shared their institution's clinical experience highlighting the utility and safety of VCE. Through technological research and development TNE has become an essential tool for the pediatric gastroenterologist. Bose and Pitman presented the historical and technical aspects of TNE with practical clinical guidance, while Tran et al. expanded further and delivered their group's expert opinion. Having appropriate minimal invasive tools to enable visualization of previously difficult-to-access areas of the intestine, like the small intestine, is reshaping the diagnostic approach while broadening access to care, as highlighted in the enteroscopy review by Hoskins. These tools are particularly impactful in children, where the risks and logistical challenges of sedation are usually eliminated.

## Diagnostic precision through endoscopy

At the same time, the field is undergoing a transition from macroscopic visualization to enhanced diagnostic precision at both microstructural and functional levels. Dalpiaz et al. reviewed the pediatric confocal laser endomicroscopy experience, which has the potential to offer real-time, *in vivo* histologic assessment, narrowing the gap between endoscopy and pathology for children. Complementing this, functional luminal imaging planimetry (EndoFLIP) provides quantitative assessment of luminal geometry and compliance as explained by Berson et al., introducing an objective dimension to disorders traditionally defined by subjective symptoms, like eosinophilic esophagitis. Together, these technologies signal a shift toward precision diagnostics, where structural, cellular, and functional data can be integrated to refine disease characterization and guide management.

## Advances in interventional endoscopy

Parallel to these diagnostic advances is the continued expansion of therapeutic endoscopy in pediatric practice. Techniques such as endoscopic ultrasound (EUS) and endoscopic retrograde cholangiopancreatography (ERCP), once largely confined to adult populations, are increasingly being adapted for children. Zhang and Deng reported their center's pediatric EUS experience with safe and effective results. This was bolstered by a larger dataset from Roy et al. that showcased the diversity of pediatric EUS use. The emergence of disposable duodenoscopes, as reviewed by Khemka et al. further addresses long-standing concerns regarding infection transmission during ERCP, while also simplifying reprocessing logistics for pediatric patients. Collectively, these developments are redefining the therapeutic capabilities of the pediatric endoscopist and, in many cases, reducing the need for surgical intervention.

## Artificial intelligence integration

Overlaying these innovations is the emergence of artificial intelligence (AI) in pediatric endoscopy. Machine learning algorithms applied to image analysis hold the potential to enhance lesion detection, reduce interobserver variability, and standardize reporting. This is showcased by Stewart et al. and includes consideration for the educational and ethical aspects of AI. In pediatrics, where expertise may be concentrated in specialized centers and datasets are comparatively limited, AI offers the promise of augmenting clinical decision-making and delivering universal access to high-level interpretive support. At the same time, the integration of AI into pediatric practice raises important considerations, including the need for robust validation in diverse pediatric populations, transparency in algorithm design, and thoughtful incorporation into clinical workflows.

## Conclusions

Several cross-cutting themes emerge across these contributions. First, the continued miniaturization and adaptation of technologies for pediatric use remain essential for broader implementation. Second, there exists a persistent gap between adult innovation and pediatric application, underscoring the need for dedicated pediatric studies rather than reliance on extrapolation. Third, the increasing complexity of endoscopic tools and data streams has important implications for training, necessitating new educational frameworks to prepare the next generation of pediatric gastroenterologists despite the lower volume of pediatric cases. Finally, issues of access and equity must be addressed to ensure that these advances do not widen existing disparities in care.

Looking ahead, the future of pediatric gastrointestinal management will likely be defined by the integration of these modalities. The convergence of advanced imaging, functional assessment, and AI has the potential to move the field toward robust personalized care. Achieving this vision will require collaborative, multicenter efforts to generate high-quality pediatric data, establish standardized outcome measures, and evaluate cost-effectiveness. Equally important will be maintaining a focus on safety, feasibility, and patient-centered outcomes.

This Research Topic highlights a field in rapid transition as it accelerates beyond traditional boundaries of visualization toward a more comprehensive, precise, and technologically integrated approach to care. As these innovations continue to evolve, they offer an opportunity not only to improve diagnostic and therapeutic capabilities, but also to fundamentally redefine how we understand and manage gastrointestinal disease in children. In this context, the work presented here serves both as a reflection of current progress and as a catalyst for future discovery.

